# *H*_*∞*_ Cluster Synchronization for a Class of Neutral Complex Dynamical Networks with Markovian Switching

**DOI:** 10.1155/2014/785706

**Published:** 2014-04-27

**Authors:** Xinghua Liu

**Affiliations:** Department of Auto, School of Information Science and Technology, University of Science and Technology of China, Anhui 230027, China

## Abstract

*H*_*∞*_ cluster synchronization problem for a class of neutral complex dynamical networks (NCDNs) with Markovian switching is investigated in this paper. Both the retarded and neutral delays are considered to be interval mode dependent and time varying. The concept of *H*_*∞*_ cluster synchronization is proposed to quantify the attenuation level of synchronization error dynamics against the exogenous disturbance of the NCDNs. Based on a novel Lyapunov functional, by employing some integral inequalities and the nature of convex combination, mode delay-range-dependent *H*_*∞*_ cluster synchronization criteria are derived in the form of linear matrix inequalities which depend not only on the disturbance attenuation but also on the initial values of the NCDNs. Finally, numerical examples are given to demonstrate the feasibility and effectiveness of the proposed theoretical results.

## 1. Introduction

During the past decades, the research on the complex dynamical networks (CDNs) has attracted extensive attention of scientific and engineering researchers in all fields domestic and overseas since the pioneering work of Watts and Strogatz [1]. One of the reasons is that the complex networks have extensively existed in many practical applications, such as ecosystems, the Internet, scientific citation web, biological neural networks, and large scale robotic system; see, for example, [2–4]. It should be noted that the synchronization phenomena of CDNs have been paid more attention to and intensively have been investigated in various different fields; please refer to [5–10] and references therein for more details.

Since time delay inevitably exists and has become an important issue in studying the CDNs, synchronization problems for complex networks with time delays have gained increasing research attention and considerable progress has been made; see, for example, [5–16] and references therein for more details. However, in some practical applications, past change rate of the state variables affects the dynamics of nodes in the networks. This kind of complex dynamical network is termed as neutral complex dynamical network (NCDN), which contains delays both in its states and in the derivatives of its states. There are some results about the synchronization design problem for neutral systems [17–21]. In these works, [18, 19] had studied the synchronization control for a kind of master-response setup and further extended to the case of neutral-type neural networks with stochastic perturbation. References [17, 20] had researched the synchronization problem for a class of complex networks with neutral-type coupling delays. Reference [21] had investigated the robust global exponential synchronization problem for an array of neutral-type neural networks. However, much fewer results have been proposed for neutral complex dynamical networks (NCDNs) compared with the rich results for CDNs with only discrete delays.

Recently, as a special synchronization on CDNs, cluster synchronization has been observed in biological science, distributed computation, and social contact networks. Because most of these networks have the clustering characteristic, many individuals maintain close contact with others in a same cluster, while only a few individuals link with an outside cluster. Hence, the individuals are synchronized inside the same cluster, but there is no synchronization among the clusters. Many researchers have made a lot of progress on the cluster synchronization problem; see, for example, [22–26]. In [24], cluster synchronization criteria are proposed for the coupled Josephson equation by constructing different coupling schemes. Then, in [26], a coupling scheme with cooperative and competitive weigh couplings is used to realize cluster synchronization for connected chaotic networks. In [22], cluster synchronization in an array of hybrid coupled neural networks with delays has been investigated and a new method is proposed to realize cluster synchronization by constructing a special coupling matrix. Besides, in the latest two years, cluster synchronization is considered for an array of coupled stochastic delayed neural networks by using the pinning control strategy in [23]. Linear pinning control schemes are given for cluster mixed synchronization of complex networks with community structure and nonidentical nodes in [25]. However, most of the research results in general complex networks ensure global or asymptotical synchronization, but the external disturbance is always existent, which may cause complex networks to diverge or oscillate. Therefore it is imperative to enhance the anti-interference ability of the system. To our knowledge, not much has been done for *H*_*∞*_ cluster synchronization for continuous-time complex dynamical networks with neutral time delays and Markovian switching. The purpose of this paper is to minimize this gap. In addition, due to the complexity of high-order and large-scale networks, network mode switching is also a universal phenomenon in CDNs of the actual systems, and sometimes the network has finite modes that switch from one to another with certain transition rate; then such switching can be governed by a Markovian chain. The stability and synchronization problem of complex networks and neural networks with Markovian jump parameters and delays are investigated in [15, 27–30] and references therein. Motivated by the above analysis, the *H*_*∞*_ cluster synchronization problem for a class of NCDNs with Markovian switching and mode-dependent time-varying delays is investigated in this paper. The addressed NCDNs consist of *M* modes and the networks switch from one mode to another according to a Markovian chain.

In this paper, *H*_*∞*_ cluster synchronization of the NCDNs with Markovian jump parameters is studied for the first time, which is first introduced to quantify the attenuation level of synchronization error dynamics against the exogenous disturbance of NCDNs with Markovian switching. It is assumed that the neutral and retarded delays are interval mode dependent and time varying. By utilizing a new augmented Lyapunov functional, *H*_*∞*_ cluster synchronization criteria, which depend on interval mode-dependent delays, disturbance attenuation lever, and the initial values of NCDNs, are derived based on the Lyapunov stability theory, integral matrix inequalities, and convex combination. All the proposed results are in terms of LMIs that can be solved numerically, which are proved to be effective in numerical examples.

The remainder of the paper is organized as follows.  Section 2 presents the problem and preliminaries.  Section 3 gives the main results, which are then verified by numerical examples in Section 4. The paper is concluded in Section 5.

*Notations*. The following notations are used throughout the paper. *R* ^*n*^ denotes the *n* dimensional Euclidean space and *R* ^*m*×*n*^ is the set of all *m* × *n* matrices. *X* < *Y* (*X* > *Y*), where *X* and *Y* are both symmetric matrices, meaning that *X* − *Y* is negative (positive) definite. *I* is the identity matrix with proper dimensions. For a symmetric block matrix, we use ∗ to denote the terms introduced by symmetry. *E* stands for the mathematical expectation, ||*v*|| is the Euclidean norm of vector *v*, and ||*v*|| = (*v* ^*T*^ *v*)^1/2^, while ||*A*|| is spectral norm of matrix *A* and ||*A*|| = [*λ*_max⁡_(*A* ^*T*^ *A*)]^1/2^. *λ*_max⁡(min⁡)_(*A*) is the eigenvalue of matrix *A* with maximum (minimum) real part. The Kronecker product of matrices *P* ∈ *R* ^*m*×*n*^ and *Q* ∈ *R* ^*p*×*q*^ is a matrix in *R* ^*mp*×*nq*^ which is denoted by *P* ⊗ *Q*. Let *ς* > 0 and *C*([−*ς*, 0], *R* ^*n*^) denotes the family of continuous function *φ*, from [−*ς*, 0] to *R* ^*n*^ with the norm |*φ*| = sup⁡_−*ς*≤*θ*≤0_||*φ*(*θ*)||. Matrices, if their dimensions are not explicitly stated, are assumed to have compatible dimensions for algebraic operations.

## 2. Problem Statement and Preliminaries

Given a complete probability space {*Ω*, *F*, **P**} where *Ω* is the sample space, *F* is the algebra of events and **P** is the probability measure defined on *F*. Let {*r*(*t*),  *t* ≥ 0} be a homogeneous and right-continuous Markov chain taking values in a finite state space *S* = {1,2, 3,…, *M*} with a generator *Υ* = (*γ*_*ij*_)_*M*×*M*_, *i*, *j* ∈ *S*, which is given by

(1) P(r(t+Δt)=j ∣ r(t)=i)={γijΔt+o(Δt)i≠j1+γiiΔt+o(Δt)i=j,

where Δ*t* > 0, lim⁡_Δ*t*→0_(*o*(Δ*t*)/Δ*t*) = 0, *γ*_*ij*_ ≥ 0 (*i*, *j* ∈ *S*, *i* ≠ *j*) is the transition rate from mode *i* to *j* and, for any state or mode *i* ∈ *S*, it satisfies

(2) $$\begin{array}{c} \gamma_{ii}=-\overset{M}{\underset{j=1,j\neq i}{\sum }}\gamma_{ij},\;\;\eta =\underset{i\in S}{\max}\left\{-\gamma_{ii}\right\}. \end{array}$$

Moreover, it is assumed that *r*(*t*) is irreducible and available at time *t*.

The following neutral complex dynamical network (NCDN) consisting of *N* identical nodes with Markovian jump parameters and interval time-varying delays over the space {*Ω*, *F*, **P**} is investigated in this paper:

(3) x˙k(t)−C(r(t))x˙k(t−τ(t,r(t)))  =A(r(t))xk(t)+B(r(t))xk(t−d(t,r(t)))   +∑l=1Ngkl(1)(r(t))Γ1(r(t))xl(t)   +∑l=1Ngkl(2)(r(t))Γ2(r(t))xl(t−d(t,r(t)))   +∑l=1Ngkl(3)(r(t))Γ3(r(t))x˙l(t−τ(t,r(t)))   +D(r(t))f1(xk(t))   +E(r(t))f2(xk(t−d(t,r(t))))   +F(r(t))f3(x˙k(t−τ(t,r(t))))   +Hk(r(t))ωk(t),

(4) $$\begin{array}{c} z_{k}\left(t\right)=L\left(r\left(t\right)\right)x_{k}\left(t\right), \end{array}$$

where *x*_*k*_(*t*) = (*x*_*k*1_(*t*),*x*_*k*2_(*t*),…,*x*_*kn*_(*t*))^*T*^ ∈ *R* ^*n*^ and *z*_*k*_(*t*) = (*z*_*k*1_(*t*),*z*_*k*2_(*t*),…,*z*_*kn*_(*t*))^*T*^ ∈ *R* ^*n*^ are state variable and the controlled output of the node *k* ∈ {1,2,…, *N*}, respectively. *ω*_*k*_(*t*) ∈ *R* is the exogenous disturbance input. *r*(*t*) describes the evolution of the mode. *A*(*r*(*t*)), *B*(*r*(*t*)), *C*(*r*(*t*)), *D*(*r*(*t*)), *E*(*r*(*t*)), and *F*(*r*(*t*)) ∈ *R* ^*n*×*n*^ represent the connection weight matrices and the delayed connection weight matrices with real values in mode *r*(*t*). *H*_*k*_(*r*(*t*)) ∈ *R* ^*n*^ (*k* = 1,2,…, *N*) is the disturbance matrix in mode *r*(*t*). *L*(*r*(*t*)) ∈ *R* ^*n*×*n*^ is a parametric matrix in mode *r*(*t*). In this paper, these parametric matrices of NCDN (3) and (4) are known constant matrices in certain mode *r*(*t*). *f*_1_, *f*_2_, *f*_3_ : *R* ^*n*^ → *R* ^*n*^ are continuously nonlinear vector functions which are with respect to the current state *x*(*t*), the delayed state *x*(*t* − *d*(*t*, *r*(*t*))), and the neutral delay state ${\dot{x}}_{k}\left(t-\tau \left(t,r\left(t\right)\right)\right)$. Γ_1_(*r*(*t*)) ∈ *R* ^*n*×*n*^, Γ_2_(*r*(*t*)) ∈ *R* ^*n*×*n*^, and Γ_3_(*r*(*t*)) ∈ *R* ^*n*×*n*^ represent the inner-coupling matrices linking between the subsystems in mode *r*(*t*). *G* ^(1)^(*r*(*t*)) = [*g*_*kl*_^(1)^]_*N*×*N*_, *G* ^(2)^(*r*(*t*)) = [*g*_*kl*_^(2)^]_*N*×*N*_, and *G* ^(3)^(*r*(*t*)) = [*g*_*kl*_^(3)^]_*N*×*N*_ are the coupling configuration matrices of the networks representing the coupling strength and the topological structure of the NCDNs in mode *r*(*t*), in which *g*_*kl*_^(*m*)^ is defined as follows. If there exists a connection between *k*th and *l*th (*k* ≠ *l*) nodes, then *g*_*kl*_^(*m*)^(*r*(*t*)) = *g*_*lk*_^(*m*)^(*r*(*t*)) > 0; otherwise *g*_*kl*_^(*m*)^(*r*(*t*)) = *g*_*lk*_^(*m*)^(*r*(*t*)) = 0 and

(5) gkk(m)(r(t)) =−∑l=1,l≠kNgkl(m)(r(t))=−∑l=1,l≠kNglk(m)(r(t)),       m=1,2,3;  k=1,2,…,N.

*τ*(*t*, *r*(*t*)) and *d*(*t*, *r*(*t*)) denote the mode-dependent time-varying neutral delay and retarded delay, respectively. They are assumed to satisfy

(6) $$\begin{array}{c} 0\leq \tau_{1i}\leq \tau_{i}\left(t\right)\leq \tau_{2i}\leq \bar{\tau }=\underset{i\in S}{\max}\left\{\tau_{2i}\right\},\\ {\dot{\tau }}_{i}\left(t\right)\leq \nu_{i}<1,\\ 0\leq d_{1i}\leq d_{i}\left(t\right)\leq d_{2i},\;\text{when}\;\;r\left(t\right)=i\in S, \end{array}$$

where *τ*_1*i*_, *τ*_2*i*_, *d*_1*i*_, and *d*_2*i*_ are real constant scalars and *ς* = max⁡_*i*∈*S*_{*τ*_2*i*_, *d*_2*i*_}.

The nonlinear vector functions, *f*_1_, *f*_2_, and *f*_3_, are assumed to satisfy the following sector-bounded condition [31]:

(7) [f1(x)−f1(y)−W1(1)(x−y)]T ×[f1(x)−f1(y)−W2(1)(x−y)]≤0, ∀x,y∈Rn,[f2(x)−f2(y)−W1(2)(x−y)]T ×[f2(x)−f2(y)−W2(2)(x−y)]≤0, ∀x,y∈Rn,[f3(x)−f3(y)−W1(3)(x−y)]T ×[f3(x)−f3(y)−W2(3)(x−y)]≤0, ∀x,y∈Rn,

where *W*_1_^(*l*)^ and *W*_2_^(*l*)^, *l* = 1,2, 3, are two constant matrices with *W*_2_^(*l*)^ − *W*_1_^(*l*)^ ≥ 0. Such a description of nonlinear functions has been exploited in [32–34] and is more general than the commonly used Lipschitz conditions, which would be possible to reduce the conservatism of the main results caused by quantifying the nonlinear functions via a matrix inequality technique.

For simplicity of notations, we denote *A*(*r*(*t*)), *B*(*r*(*t*)), *C*(*r*(*t*)), *D*(*r*(*t*)), *E*(*r*(*t*)), *F*(*r*(*t*)), *G* ^(*m*)^(*r*(*t*)), Γ_*m*_(*r*(*t*)), (*m* = 1,2, 3), *H*_*k*_(*r*(*t*)), and *L*(*r*(*t*)) by *A*_*i*_, *B*_*i*_, *C*_*i*_, *D*_*i*_, *E*_*i*_, *F*_*i*_, *G*_*i*_^(*m*)^, Γ_*mi*_, (*m* = 1,2, 3), *H*_*ki*_, and *L*_*i*_ for *r*(*t*) = *i* ∈ *S*. By utilizing the Kronecker product of the matrices, (3) and (4) can be written in a more compact form as

(8) x˙(t)=Aix(t)+Bix(t−di(t)) +Cix˙(t−τi(t))+DiF1(x(t)) +EiF2(x(t−di(t))) +FiF3(x˙(t−τi(t)))+Hiω(t),

(9) $$\begin{array}{c} z\left(t\right)=L_{i}x\left(t\right), \end{array}$$

where

(10) Ai=IN⊗Ai+Gi(1)⊗Γ1i,Bi=IN⊗Bi+Gi(2)⊗Γ2i,Ci=IN⊗Ci+Gi(3)⊗Γ3i,Di=IN⊗Di,  Ei=IN⊗Ei,Fi=IN⊗Fi,  Li=IN⊗Li,Hi=diag⁡{H1i,H2i,…,HNi},x(t)=col{x1(t),x2(t),…,xN(t)},x(t−di(t))=col{x1(t−di(t)),x2(t−di(t)),…,xN(t−di(t))},x˙(t−τi(t))=col{x˙1(t−τi(t)),x˙2(t−τi(t)),…,x˙N(t−τi(t))},F1(x(t))=col{f1(x1(t)),f1(x2(t)),…,f1(xN(t))},F2(x(t−di(t))) =col{f2(x1(t−di(t))),f2(x2(t−di(t))),…,    f2(xN(t−di(t)))},F3(x˙(t−τi(t))) =col{f3(x˙1(t−τi(t))),f3(x˙2(t−τi(t))),…,    f3(x˙N(t−τi(t)))},ω(t)=col{ω1(t),ω2(t),…,ωN(t)},z(t)=col{z1(t),z2(t),…,zN(t)}.

**Assumption 1 (see [22])**

The coupling matrix *G*_*i*_^(*m*)^ can be expressed in the following form:

(11) $$\begin{array}{c} G_{i}^{\left(m\right)}=\left[\begin{array}{cccc} N_{i11}^{\left(m\right)} & N_{i12}^{\left(m\right)} & \cdots & N_{i1k}^{\left(m\right)}\\ N_{i21}^{\left(m\right)} & N_{i22}^{\left(m\right)} & \cdots & N_{i2k}^{\left(m\right)}\\ \vdots & \vdots & \ddots & \vdots \\ N_{ik1}^{\left(m\right)} & N_{ik2}^{\left(m\right)} & \cdots & N_{ikk}^{\left(m\right)} \end{array}\right],\;m=1,2,3. \end{array}$$

It should be especially emphasized that we do not assume that the coupling matrix is symmetric or diagonal. However, most of the former works about network synchronization are based on symmetric or diagonal coupling matrix.

Before moving onto the main results, some definitions and lemmas are introduced below.

**Definition 2 (see [35])**

Define operator *D* : *C*([−*ς*, 0], *R* ^*n*^) → *R* ^*n*^ by *D*(*x*_*t*_) = *x*(*t*) − *Cx*(*t* − *τ*). *D* is said to be stable if the homogeneous difference equation

(12) $$\begin{array}{c} D\left(x_{t}\right)=0,\;t\geq 0,\\ x_{0}=\psi \in \left\{\phi \in C\left(\left[-\varsigma ,0\right],R^{n}\right):D\phi =0\right\} \end{array}$$

is uniformly asymptotically stable. In this paper, that is, ||*I*_*N*_ ⊗ *C*_*i*_ + *G*_*i*_^(3)^ ⊗ *C*_*i*_|| < 1.

**Definition 3 (see [36])**

Define the stochastic Lyapunov-Krasovskii function of the NCDNs (3) and (4) as *V*(*x*(*t*), *r*(*t*) = *i*,  *t* > 0) = *V*(*x*(*t*), *i*, *t*) where its infinitesimal generator is defined as

(13) ΓV(x(t),i,t) =lim⁡Δt→0⁡1Δt[E{V(x(t+Δt),r(t+Δt),           t+Δt) ∣ x(t)=x, r(t)=i}      −V(x(t),i,t)] =∂∂tV(x(t),i,t)+∂∂xV(x(t),i,t)x˙(t)  +∑j=1NπijV(x(t),j,t).

**Definition 4 (see [26])**

A network with *N* nodes realizes cluster synchronization if the *N* nodes are split into several clusters, such as {(1,2,…, *m*_1_), (*m*_1_ + 1, *m*_1_ + 2,…, *m*_2_),…, (*m*_*k*−1_ + 1, *m*_*k*−1_ + 2,…, *m*_*k*_), *m*_0_ = 0, *m*_*k*_ = *N*, *m*_*j*−1_ < *m*_*j*_}, and the nodes in the same cluster synchronize with one another (i.e., for the states *x*_*i*_(*t*) and *x*_*j*_(*t*) of arbitrary nodes *i* and *j* in the same cluster, lim⁡_*t*→*∞*_||*x*_*i*_(*t*) − *x*_*j*_(*t*)|| = 0 holds). The set

(14) S={x=(x1(s),x2(s),…,xN(s)):x1(s)=x2(s)=⋯  =xm1(s),xm1+1(s)=xm1+2(s)=⋯  =xm2(s),…,xmk−1+1(s)=xmk−1+2(s)=⋯=xmk(s)}

is called the cluster synchronization manifold.

**Lemma 5 (see [37])**

Let *G* be an *N* × *N* matrix in the set *T*(*R*, *K*), where *R* denotes a ring and *T*(*R*, *K*) = {the set of matrices with entries *R* such that the sum of the entries in each row is equal to *K* for some *K* ∈ *R*}. Then the (*N* − 1)×(*N* − 1) matrix *X* satisfies *MG* = *XM*, where *X* = *MG* *J*,

(15) M=[1−11−11−1⋱1−1](N−1)×N,J=[111⋯1011⋯1⋱100⋯1100⋯01000⋯0]N×(N−1).

Furthermore, the matrix *X* can be rewritten explicitly as follows:

(16) Xp,q=∑k=1q(Gp,k−Gp+1,k), for p,q∈{1,2,…,N−1}.

**Lemma 6**

Under Assumption 1, the (*N* − *k*)×(*N* − *k*) matrix *X*_*i*_^(*m*)^ satisfies $\tilde{M}G_{i}^{\left(m\right)}=X_{i}^{\left(m\right)}\tilde{M}$, *m* = 1,2, 3, where

(17) Ni(m)=[Ni11(m)Ni22(m)⋱Nikk(m)]N×N,M~=[M1M2⋱Mk](N−k)×N,J~=[J1J2⋱Jk]N×(N−k).

And $X_{i}^{\left(m\right)}=\tilde{M}N_{i}^{\left(m\right)}\tilde{J}$, *N*_*pp*_^(*m*)^ ∈ *R* ^*m*_*p*_×*m*_*p*_^, *M*_*p*_ ∈ *R* ^(*m*_*p*_−1)×*m*_*p*_^, *J*_*p*_ ∈ *R* ^*m*_*p*_×(*m*_*p*_−1)^, and *p* = 1,2,…, *k*.

**Proof**

From Assumption 1 and Lemma 5, it can be easily obtained that

(18) M~Gi(m) =[M1M2⋱Mk]  ×[Ni11(m)Ni12(m)⋯Ni1k(m)Ni21(m)Ni22(m)⋯Ni2k(m)⋮⋮⋱⋮Nik1(m)Nik2(m)⋯Nikk(m)] =[M1Ni11(m)M2Ni22(m)⋱MkNikk(m)] =[M1Ni11(m)J1M1M2Ni22(m)J2M2⋱MkNikk(m)JkMk] =M~Ni(m)J~M~=Xi(m)M~.

This completes the proof.

**Lemma 7 (see [22])**

*x* ∈ *S* if and only if *E*{||**M** *x*(*t*)||^2^} = 0, *t* → *∞*, where $M=\tilde{M}⊗I_{N}$.

**Proof**

Consider

(19) E{||Mx(t)||2}=E{∑l=1m1−1||xl(t)−xl+1(t)||2  +∑l=m1+1m2−1||xl(t)−xl+1(t)||2+⋯  +∑l=mk−1+1mk−1||xl(t)−xl+1(t)||2}.

By Definition 4, it completes the proof.

**Definition 8**

The neutral complex dynamical networks (3) and (4) are *H*_*∞*_ cluster synchronization with a disturbance attenuation *δ* and symmetric positive matrix *Y* > 0, if the following condition is satisfied:

(20) ∫0∞||Mz(t)||2dt≤δ2{∫0∞||ω(t)||2dt+xT(0)Yx(0)}.

The index *δ* is called disturbance attenuation and used to quantify the attenuation level of synchronization error dynamics against exogenous disturbances. It is noticed that (20) depends not only on the attenuation level but also on the initial values of complex networks.

**Lemma 9 (see [38])**

Given matrices *A*, *B*, *C*, and *D* with appropriate dimensions and scalar *α*, by the definition of the Kronecker product, the following properties hold:

(21) $$\begin{array}{c} \left(\alpha A\right)⊗B=A⊗\left(\alpha B\right),\\ \left(A+B\right)⊗C=A⊗C+B⊗C,\\ \left(A⊗B\right)\left(C⊗D\right)=\left(AC\right)⊗\left(BD\right),\\ {\left(A⊗B\right)}^{T}=A^{T}⊗B^{T}. \end{array}$$

**Lemma 10 (see [39, 40])**

For any constant matrix *H* = *H* ^*T*^ > 0 and scalars *τ*_2_ > *τ*_1_ > 0 such that the following integrations are well defined, then

(a) (22) −(τ2−τ1)∫t−τ2t−τ1xT(s)Hx(s)ds  ≤−[∫t−τ2t−τ1xT(s)ds]H[∫t−τ2t−τ1x(s)ds],
(b) (23) −12(τ22−τ12)∫−τ2−τ1∫t+θtxT(s)Hx(s)ds dθ  ≤−[∫−τ2−τ1∫t+θtxT(s)ds dθ]   ×H[∫−τ2−τ1∫t+θtx(s)ds dθ].

**Lemma 11 (see [41])**

Supposing that 0 ≤ *τ*_*m*_ ≤ *τ*(*t*) ≤ *τ*_*M*_, *Ξ*_1_, *Ξ*_2_, and *Ω* are constant matrices of appropriate dimensions, then

(24) (τ(t)−τm)Ξ1+(τM−τ(t))Ξ2+Ω<0

if and only if (*τ*_*M*_ − *τ*_*m*_)*Ξ*_1_ + *Ω* < 0 and (*τ*_*M*_ − *τ*_*m*_)*Ξ*_2_ + *Ω* < 0 hold.

## 3. Main Results

In this section, sufficient conditions are presented to ensure *H*_*∞*_ cluster synchronization for the neutral complex dynamical network (NCDN) (3) and (4).

### 3.1. *H*_*∞*_ Cluster Synchronization Analysis

**Theorem 12**

Given the transition rate matrix *Υ*, the initial positive definite matrix *Y* = *Y* ^*T*^ > 0, constant scalars *τ*_1*i*_, *τ*_2*i*_, *ν*_*i*_, *d*_1*i*_, *d*_2*i*_, and *τ*_*mi*_, *d*_*mi*_ satisfying *τ*_1*i*_ < *τ*_*mi*_ < *τ*_2*i*_, *d*_1*i*_ < *d*_*mi*_ < *d*_2*i*_, respectively, the NCDN systems (3) and (4) with sector-bounded condition (7) are *H*_*∞*_ cluster synchronization with a disturbance attenuation lever *δ* if ||(*I*_*N*_ + *G*_*i*_^(3)^) ⊗ *C*_*i*_|| < 1 and there exist (*N* − *k*)*n* × (*N* − *k*)*n* symmetric positive matrices *P*_*i*_ > 0, (*i* ∈ *S*), *Q*_*j*_ > 0, (*j* = 1,2,…, 6), *R*_*k*_ > 0, (*k* = 1,2,…, 7), *T*_*l*_ > 0, *U*_*m*_ > 0, and *V*_*n*_ > 0, (*l*, *m*, *n* = 1,2,…, 6) for any scalars *ϵ*_1_, *ϵ*_2_, *ϵ*_3_ > 0 such that the following linear matrix inequalities hold:

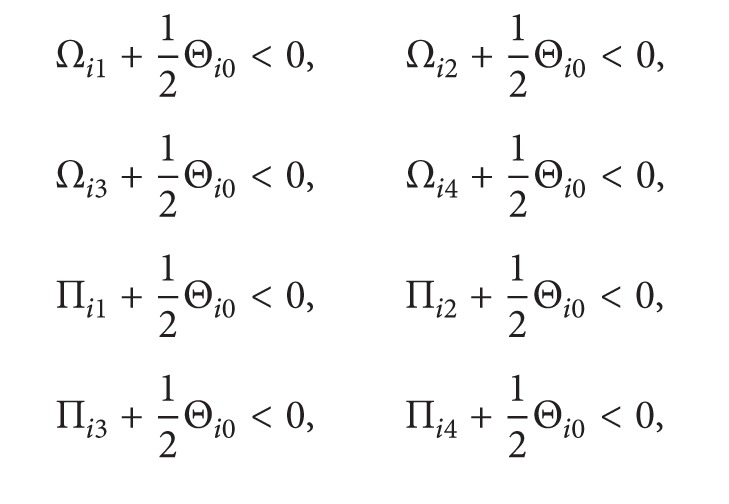
(25)

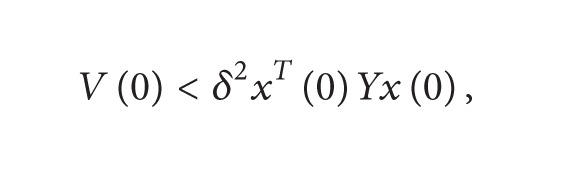
(26)
where

(27) Θi0=∑m=130EmΦmEmT+L(✠)+ΛTJΛ−(E1−E3) ×U1(E1T−E3T)−(E1−E16)U4(E1T−E16T) −(τ1iE1−E10)V1(τ1iE1T−E10T) −[(τmi−τ1i)E1−E13]V2[(τmi−τ1i)E1T−E13T] −[(τ2i−τmi)E1−E14]V3[(τ2i−τmi)E1T−E14T] −(d1iE1−E22)V4(d1iE1T−E22T) −[(dmi−d1i)E1−E25]V5[(dmi−d1i)E1T−E25T] −[(d2i−dmi)E1−E26]V6[(d2i−dmi)E1T−E26T],

where *E*_*i*_  {*i* = 1,2,…, 30} are block entry matrices; that is,

(28) $$\begin{array}{c} E_{4}^{T}=\left[\begin{array}{cccccccccccccccccccccccccccccc} 0 & 0 & 0 & I & 0 & 0 & 0 & 0 & 0 & 0 & 0 & 0 & 0 & 0 & 0 & 0 & 0 & 0 & 0 & 0 & 0 & 0 & 0 & 0 & 0 & 0 & 0 & 0 & 0 & 0 \end{array}\right]. \end{array}$$

*L* is a linear operator on real square matrices by

(29) L(A)=A+AT, ∀A∈Rn×n,J=η(τ¯−τ1i)R1+R2+R5+τ1i2U1+d1i2U4+(τmi−τ1i)2U2+(τ2i−τmi)2U3+(dmi−d1i)2U5+(d2i−dmi)2U6+τ1i44V1+d1i44V4+(τmi2−τ1i2)24V2+(τ2i2−τmi2)24V3+(dmi2−d1i2)24V5+(d2i2−dmi2)24V6,Λ=(Ai⊗+Xi(1)→)E1T+(Ci⊗+Xi(3)→)E6T +(Bi⊗+Xi(2)→)E15T+Di⊗E27T+Ei⊗E28T +Fi⊗E29T+MHiE30T,✠=E1(PiDi⊗+ϵ1W1(1)T+ϵ1W2(1)T)E27T+E1PiDi⊗E28T+E1PiFi⊗E29T+E1PiHiE30T+E15(ϵ2W1(2)T+ϵ2W2(2)T)E28T+E6(ϵ3W1(3)T+ϵ3W2(3)T)E29T,Φ1=L[Pi(Ai⊗+Bi⊗+Ci⊗+Xi(1)→+Xi(2)→+Xi(3)→) −ϵ1W1(1)TW2(1)]+∑j∈SγijPj+Q1+Q4+τ1i2T1+d1i2T4+(τmi−τ1i)2T2+(τ2i−τmi)2T3+(dmi−d1i)2T5+(d2i−dmi)2T6+Li⊗TLi⊗,Φ3=Q2−R1,  Φ4=Q4−R2,Φ5=−Q3,  Φ6=−(1−νi)R1−ϵ3W1(3)TW2(3),Φ7=R1+R3−R2,  Φ8=R4−R3,Φ9=−R4,  Φ10=−T1,Φ15=−ϵ2W1(2)TW2(2),  Φ16=Q5−Q4,Φ17=Q6−Q5,  Φ18=−Q6,Φ19=R6−R5,  Φ20=R7−R6,Φ21=−R7,  Φ27=−2ϵ1I,Φ28=−2ϵ2I,  Φ29=−2ϵ3I,Φ30=−δ2I,Φm=0, (m=2,11,12,13,14,23,24,25,26),Ai⊗=IN−k⊗Ai,  Bi⊗=IN−k⊗Bi,Ci⊗=IN−k⊗Ci,  Di⊗=IN−k⊗Di,Ei⊗=IN−k⊗Ei,  Fi⊗=IN−k⊗Fi,Li⊗=IN−k⊗Li,Xi(m)→=Xi(m)⊗Γmi, (m=1,2,3),Ωi1=−E11T2E11T−2(E13−E11)T2(E13T−E11T) −(E3−E2)U2(E3T−E2T)−2(E2−E4) ×U2(E2T−E4T)−E14T3E14T −(E4−E5)U3(E4T−E5T),Ωi2=−2E11T2E11T−(E13−E11)T2(E13T−E11T) −2(E3−E2)U2(E3T−E2T)−(E2−E4) ×U2(E2T−E4T)−E14T3E14T −(E4−E5)U3(E4T−E5T),Ωi3=−2E12T3E12T−(E14−E12)T3(E14T−E12T) −(E4−E2)U3(E4T−E2T)−2(E2−E5) ×U3(E2T−E5T)−E13T2E13T −(E3−E4)U2(E3T−E4T),Ωi4=−E12T3E12T−2(E14−E12)T3(E14T−E12T) −2(E4−E2)U3(E4T−E2T) −(E2−E5)U3(E2T−E5T) −E13T2E13T−(E3−E4)U2(E3T−E4T),Πi1=−E23T5E23T−2(E25−E23)T5(E25T−E23T) −(E16−E15)U5(E16T−E15T) −2(E15−E17)U5(E15T−E17T) −E26T6E26T−(E17−E18)U6(E17T−E18T),Πi2=−2E23T5E23T−(E25−E23)T5(E25T−E23T) −2(E16−E15)U5(E16T−E15T) −(E15−E17)U5(E15T−E17T) −E26T6E26T−(E17−E18)U6(E17T−E18T),Πi3=−2E24T6E24T−(E26−E24)T6(E26T−E24T) −(E17−E15)U6(E17T−E15T) −2(E15−E18)U6(E15T−E18T)−E25T5E25T −(E16−E17)U5(E16T−E17T),Πi4=−E24T6E24T−2(E26−E24)T6(E26T−E24T) −2(E17−E15)U6(E17T−E15T) −(E15−E18)U6(E15T−E18T)−E25T5E25T −(E16−E17)U5(E16T−E17T),V(0)=xT(0)MTPiMx(0)+∑k=26Vk(0),V2(0)=∫−τ1i0xT(s)MTQ1Mx(s)ds+∫−τmi−τ1ixT(s)MTQ2Mx(s)ds+∫−τ2i−τmixT(s)MTQ3Mx(s)ds+∫−d1i0xT(s)MTQ4Mx(s)ds+∫−dmi−d1ixT(s)MTQ5Mx(s)ds+∫−d2i−dmixT(s)MTQ6Mx(s)ds,V3(0)=∫−τi(t)−τ1ix˙T(s)MTR1Mx˙(s)ds+∫−τ1i0x˙T(s)MTR2Mx˙(s)ds+∫−τmi−τ1ix˙T(s)MTR3Mx˙(s)ds+∫−τ2i−τmix˙T(s)MTR4Mx˙(s)ds+∫−d1i0x˙T(s)MTR5Mx˙(s)ds+∫−dmi−d1ix˙T(s)MTR6Mx˙(s)ds+∫−d2i−dmix˙T(s)MTR7Mx˙(s)ds,V4(0)=∫−τ1i0∫θ0τ1ixT(s)MTT1Mx(s)ds dθ +∫−τmi−τ1i∫θ0(τmi−τ1i)xT(s)MTT2Mx(s)ds dθ +∫−τ2i−τmi∫θ0(τ2i−τmi)xT(s)MTT3Mx(s)ds dθ +∫−d1i0∫θ0d1ixT(s)MTT4Mx(s)ds dθ +∫−dmi−d1i∫θ0(dmi−d1i)xT(s)MTT5Mx(s)ds dθ +∫−d2i−dmi∫θ0(d2i−dmi)xT(s)MTT6Mx(s)ds dθ,V5(0)=∫−τ1i0∫θ0τ1ix˙T(s)MTU1Mx˙(s)ds dθ +∫−τmi−τ1i∫θ0(τmi−τ1i)x˙T(s)MTU2Mx˙(s)ds dθ +∫−τ2i−τmi∫θ0(τ2i−τmi)x˙T(s)MTU3Mx˙(s)ds dθ +∫−d1i0∫θ0d1ix˙T(s)MTU4Mx˙(s)ds dθ +∫−dmi−d1i∫θ0(dmi−d1i)x˙T(s)MTU5Mx˙(s)ds dθ +∫−d2i−dmi∫θ0(d2i−dmi)x˙T(s)MTU6Mx˙(s)ds dθ +∫−τ¯−τ1i∫θ0ηx˙T(s)MTR1Mx˙(s)ds dθ,V6(0)=∫−τ1i0∫θ0∫λ0τ1i22x˙T(s)MTV1Mx˙(s)ds dλ dθ +∫−τmi−τ1i∫θ0∫λ0τmi2−τ1i22x˙T(s)MTV2Mx˙(s)ds dλ dθ +∫−τ2i−τmi∫θ0∫λ0τ2i2−τmi22x˙T(s)MTV3Mx˙(s)ds dλ dθ +∫−d1i0∫θ0∫λ0d1i22x˙T(s)MTV4Mx˙(s)ds dλ dθ +∫−dmi−d1i∫θ0∫λ0dmi2−d1i22x˙T(s)MTV5Mx˙(s)ds dλ dθ +∫−d2i−dmi∫θ0∫λ0d2i2−dmi22x˙T(s)MTV6Mx˙(s)ds dλ dθ.

**Proof**

Construct the Lyapunov functional candidate as follows:

(30) $$\begin{array}{c} V\left(x\left(t\right),i,t\right)=\overset{6}{\underset{k=1}{\sum }}V_{k}\left(x\left(t\right),i,t\right), \end{array}$$

where

(3.1) V1(x(t),i,t)=xT(t)MTPiMx(t),V2(x(t),i,t)=∫t−τ1itxT(s)MTQ1Mx(s)ds +∫t−τmit−τ1ixT(s)MTQ2Mx(s)ds +∫t−τ2it−τmixT(s)MTQ3Mx(s)ds +∫t−d1itxT(s)MTQ4Mx(s)ds +∫t−dmit−d1ixT(s)MTQ5Mx(s)ds +∫t−d2it−dmixT(s)MTQ6Mx(s)ds,V3(x(t),i,t)=∫t−τi(t)t−τ1ix˙T(s)MTR1Mx˙(s)ds+∫t−τ1itx˙T(s)MTR2Mx˙(s)ds+∫t−τmit−τ1ix˙T(s)MTR3Mx˙(s)ds+∫t−τ2it−τmix˙T(s)MTR4Mx˙(s)ds+∫t−d1itx˙T(s)MTR5Mx˙(s)ds+∫t−dmit−d1ix˙T(s)MTR6Mx˙(s)ds+∫t−d2it−dmix˙T(s)MTR7Mx˙(s)ds,V4(x(t),i,t)=∫−τ1i0∫t+θtτ1ixT(s)MTT1Mx(s)ds dθ +∫−τmi−τ1i∫t+θt(τmi−τ1i)xT(s)MTT2Mx(s)ds dθ +∫−τ2i−τmi∫t+θt(τ2i−τmi)xT(s)MTT3Mx(s)ds dθ +∫−d1i0∫t+θtd1ixT(s)MTT4Mx(s)ds dθ +∫−dmi−d1i∫t+θt(dmi−d1i)xT(s)MTT5Mx(s)ds dθ +∫−d2i−dmi∫t+θt(d2i−dmi)xT(s)MTT6Mx(s)ds dθ,V5(x(t),i,t)=∫−τ1i0∫t+θtτ1ix˙T(s)MTU1Mx˙(s)ds dθ +∫−τmi−τ1i∫t+θt(τmi−τ1i)x˙T(s)MTU2Mx˙(s)ds dθ +∫−τ2i−τmi∫t+θt(τ2i−τmi)x˙T(s)MTU3Mx˙(s)ds dθ +∫−d1i0∫t+θtd1ix˙T(s)MTU4Mx˙(s)ds dθ +∫−dmi−d1i∫t+θt(dmi−d1i)x˙T(s)MTU5Mx˙(s)ds dθ +∫−d2i−dmi∫t+θt(d2i−dmi)x˙T(s)MTU6Mx˙(s)ds dθ +∫−τ¯−τ1i∫t+θtηx˙T(s)MTR1Mx˙(s)ds dθ,V6(x(t),i,t)=∫−τ1i0∫θ0∫t+λtτ1i22x˙T(s)MTV1Mx˙(s)ds dλ dθ   +∫−τmi−τ1i∫θ0∫t+λtτmi2−τ1i22x˙T(s)MTV2Mx˙(s)ds dλ dθ +∫−τ2i−τmi∫θ0∫t+λtτ2i2−τmi22x˙T(s)MTV3Mx˙(s)ds dλ dθ +∫−d1i0∫θ0∫t+λtd1i22x˙T(s)MTV4Mx˙(s)ds dλ dθ +∫−dmi−d1i∫θ0∫t+λtdmi2−d1i22x˙T(s)MTV5Mx˙(s)ds dλ dθ +∫−d2i−dmi∫θ0∫t+λtd2i2−dmi22x˙T(s)MTV6Mx˙(s)ds dλ dθ.

By the structure of **M** and by Lemmas 6 and 9, we obtain the following equalities:

(3.1) M(IN⊗Ai)=(IN−k⊗Ai)M=Ai⊗M,M(IN⊗Bi)=(IN−k⊗Bi)M=Bi⊗M,M(IN⊗Ci)=(IN−k⊗Ci)M=Ci⊗M,M(IN⊗Di)=(IN−k⊗Di)M=Di⊗M,M(IN⊗Ei)=(IN−k⊗Ei)M=Ei⊗M,M(IN⊗Fi)=(IN−k⊗Fi)M=Fi⊗M,M(Gi(m)⊗Γmi)=(M~⊗IN)(Gi(m)⊗Γmi)=(M~Gi(m))⊗Γmi=(Xi(m)M~)⊗Γmi=(Xi(m)⊗Γmi)(M~⊗IN)=Xi(m)→M, m=1,2,3,M(IN⊗Li)=(IN−k⊗Li)M=Li⊗M.

Taking Γ as its infinitesimal generator along the trajectory of (8), we obtain the following from Definition 3 and (30)–(3.1):

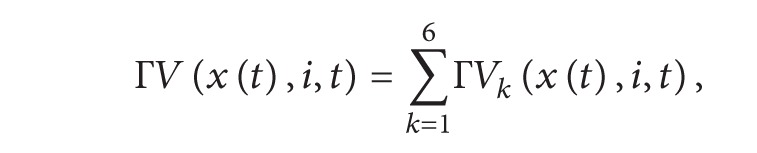
(33)

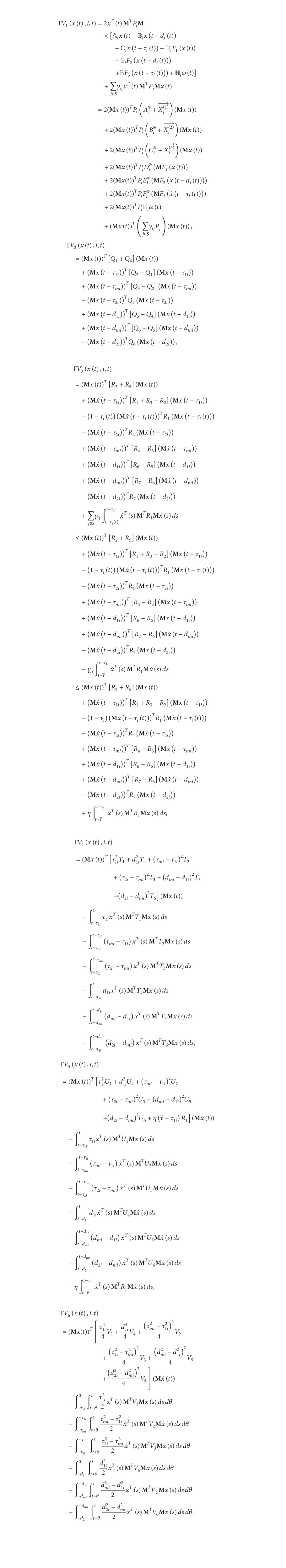
(34)
Define

(35) ξ(t)=col{Mx(t) Mx(t−τi(t)) Mx(t−τ1i)    Mx(t−τmi) Mx(t−τ2i) Mx˙(t−τi(t))    Mx˙(t−τ1i) Mx˙(t−τmi) Mx˙(t−τ2i)    M∫t−τ1itx(s)ds M∫t−τi(t)t−τ1ix(s)ds    M∫t−τ2it−τi(t)x(s)ds M∫t−τmit−τ1ix(s)ds    M∫t−τ2it−τmix(s)ds Mx(t−di(t)) Mx(t−d1i)    Mx(t−dmi) Mx(t−d2i) Mx˙(t−d1i)    Mx˙(t−dmi) Mx˙(t−d2i) M∫t−d1itx(s)ds    M∫t−di(t)t−d1ix(s)ds M∫t−d2it−di(t)x(s)ds    M∫t−dmit−d1ix(s)ds M∫t−d2it−dmix(s)ds MF1(x(t))     MF2(x(t−di(t))) MF3(x˙(t−τi(t))) ω(t)}.

According to (7), we can obtain the following inequalities for any *ϵ*_1_, *ϵ*_2_, *ϵ*_3_ > 0:

(36) ϵ1[MF1(x(t))]T[MF1(x(t))]  =ϵ1∑j=1N−1[f1(xj(t))−f1(xj+1(t))]T   ×[f1(xj(t))−f1(xj+1(t))]  ≤ϵ1∑j=1N−1{[f1(xj(t))−f1(xj+1(t))]TW2(1)   ×(xj(t)−xj+1(t))+(xj(t)−xj+1(t))T   ×W1(1)T[f1(xj(t))−f1(xj+1(t))]   −(xj(t)−xj+1(t))TW1(1)TW2(1)×(xj(t)−xj+1(t))}  =ϵ1[MF1(x(t))]TW2(1)[M(x(t))]   +ϵ1[M(x(t))]TW1(1)T[MF1(x(t))]   −ϵ1[M(x(t))]TW1(1)TW2(1)[M(x(t))],ϵ2[MF2(x(t−di(t)))]T[MF2(x(t−di(t)))]  ≤ϵ2[MF2(x(t−di(t)))]TW2(2)[Mx(t−di(t))]   +ϵ2[M(x(t−di(t)))]TW1(2)T[MF2(x(t−di(t)))]   −ϵ2[M(x(t−di(t)))]TW1(2)TW2(2)   ×[M(x(t−di(t)))],ϵ3[MF3(x˙(t−τi(t)))]T[MF3(x˙(t−τi(t)))]≤ϵ3[MF3(x˙(t−τi(t)))]TW2(3)[M(x˙(t−τi(t)))] +ϵ3[M(x˙(t−τi(t)))]TW1(3)T[MF3(x˙(t−τi(t)))] −ϵ3[M(x˙(t−τi(t)))]TW1(3)TW2(3)[M(x˙(t−τi(t)))].

From (33) and (36), we have

(37) ΓV(x(t),i,t)  ≤∑k=16ΓVk(x(t),i,t)   −2ϵ1[MF1(x(t))]T[MF1(x(t))]   +2ϵ1[MF1(x(t))]TW2(1)(Mx(t))   +2ϵ1(Mx(t))TW1(1)T[MF1(x(t))]   −2ϵ1[Mx(t)]TW1(1)TW2(1)[Mx(t)]   −2ϵ2[MF2(x(t−di(t)))]T[MF2(x(t−di(t)))]   +2ϵ2[MF2(x(t−di(t)))]TW2(2)(Mx(t−di(t)))   +2ϵ2(Mx(t−di(t)))TW1(2)T[MF2(x(t−di(t)))]   −2ϵ2[Mx(t−di(t))]TW1(2)TW2(2)[Mx(t−di(t))]   −2ϵ3[MF3(x˙(t−τi(t)))]T[MF3(x˙(t−τi(t)))]   +2ϵ3[MF3(x˙(t−τi(t)))]TW2(3)(Mx˙(t−τi(t)))   +2ϵ3(Mx˙(t−τi(t)))TW1(3)T[MF3(x˙(t−τi(t)))]   −2ϵ3[Mx˙(t−τi(t))]TW1(3)TW2(3)[Mx˙(t−τi(t))].

Noticing (a) of Lemma 10, then

(38) −∫t−τ1itτ1ixT(s)MTT1Mx(s)ds  ≤−[M∫t−τ1itx(s)ds]TT1[M∫t−τ1itx(s)ds]  =−ξT(t)E10T1E10Tξ(t),−∫t−τ1itτ1ix˙T(s)MTU1Mx˙(s)ds  ≤−ξT(t)(E1−E3)U1(E1T−E3T)ξ(t),−∫t−d1itd1ixT(s)MTT4Mx(s)ds≤−ξT(t)E22T4E22Tξ(t),−∫t−d1itd1ix˙T(s)MTU4Mx˙(s)ds  ≤−ξT(t)(E1−E16)U4(E1T−E16T)ξ(t).

Noticing (b) of Lemma 10, then

(39) −∫−τ1i0∫t+θtτ1i22x˙T(s)MTV1Mx˙(s)ds dθ  ≤−ξT(t)(τ1iE1−E10)V1(τ1iE1T−E10T)ξ(t),−∫−τmi−τ1i∫t+θtτmi2−τ1i22x˙T(s)MTV2Mx˙(s)ds dθ  ≤−ξT(t)[(τmi−τ1i)E1−E13]×V2[(τmi−τ1i)E1T−E13T]ξ(t),−∫−τ2i−τmi∫t+θtτ2i2−τmi22x˙T(s)MTV3Mx˙(s)ds dθ  ≤−ξT(t)[(τ2i−τmi)E1−E14]   ×V3[(τ2i−τmi)E1T−E14T]ξ(t),−∫−d1i0∫t+θtd1i22x˙T(s)MTV4Mx˙(s)ds dθ  ≤−ξT(t)(d1iE1−E22)V4(d1iE1T−E22T)ξ(t),−∫−dmi−d1i∫t+θtdmi2−d1i22x˙T(s)MTV5Mx˙(s)ds dθ  ≤−ξT(t)[(dmi−d1i)E1−E25]   ×V5[(dmi−d1i)E1T−E25T]ξ(t),−∫−d2i−dmi∫t+θtd2i2−dmi22x˙T(s)MTV6Mx˙(s)ds dθ  ≤−ξT(t)[(d2i−dmi)E1−E26]   ×V6[(d2i−dmi)E1T−E26T]ξ(t).

If *τ*_*i*_(*t*)∈[*τ*_1*i*_, *τ*_*mi*_] and *d*_*i*_(*t*)∈[*d*_1*i*_, *d*_*mi*_], let

(40) λ1i(t)=τi(t)−τ1iτmi−τ1i,  κ1i(t)=di(t)−d1idmi−d1i.

Then the following is held from (a) of Lemma 10:

(41) −∫t−τmit−τ1i(τmi−τ1i)xT(s)MTT2Mx(s)ds=−{∫t−τi(t)t−τ1i+∫t−τmit−τi(t)}(τmi−τ1i)xT(s)MTT2Mx(s)ds=−(τmi−τi(t))∫t−τi(t)t−τ1ixT(s)MTT2Mx(s)ds −(τi(t)−τ1i)∫t−τi(t)t−τ1ixT(s)MTT2Mx(s)ds −(τmi−τi(t))∫t−τmit−τi(t)xT(s)MTT2Mx(s)ds −(τi(t)−τ1i)∫t−τmit−τi(t)xT(s)MTT2Mx(s)ds≤−ξT(t)E11T2E11Tξ(t) −(1−λ1i(t))ξT(t)E11T2E11Tξ(t) −ξT(t)(E13−E11)T2(E13T−E11T)ξ(t) −λ1i(t)ξT(t)(E13−E11)T2(E13T−E11T)ξ(t).

Similarly,

(42) −∫t−τmit−τ1i(τmi−τ1i)x˙T(s)MTU2Mx˙(s)ds=−{∫t−τi(t)t−τ1i+∫t−τmit−τi(t)}(τmi−τ1i)x˙T(s)MTT2Mx˙(s)ds≤−ξT(t)(E3−E2)U2(E3T−E2T)ξ(t) −(1−λ1i(t))ξT(t)(E3−E2)U2(E3T−E2T)ξ(t) −ξT(t)(E2−E4)U2(E2T−E4T)ξ(t) −λ1i(t)ξT(t)(E2−E4)U2(E2T−E4T)ξ(t),−∫t−dmit−d1i(dmi−d1i)xT(s)MTT5Mx(s)ds=−{∫t−di(t)t−d1i+∫t−dmit−di(t)}(dmi−d1i)xT(s)MTT5Mx(s)ds≤−ξT(t)E23T5E23Tξ(t)−(1−κ1i(t))ξT(t)E23T5E23Tξ(t) −ξT(t)(E25−E23)T5(E25T−E23T)ξ(t) −κ1i(t)ξT(t)(E25−E23)T5(E25T−E23T)ξ(t),−∫t−dmit−d1i(dmi−d1i)x˙T(s)MTU5Mx˙(s)ds=−{∫t−di(t)t−d1i+∫t−dmit−di(t)}(dmi−d1i)x˙T(s)MTU5Mx˙(s)ds≤−ξT(t)(E16−E15)U5(E16T−E15T)ξ(t) −(1−κ1i(t))ξT(t)(E16−E15)U5(E16T−E15T)ξ(t) −ξT(t)(E15−E17)U5(E15T−E17T)ξ(t)−κ1i(t)ξT(t) ×(E15−E17)U5(E15T−E17T)ξ(t).

Considering

(43) −∫t−τ2it−τmi(τ2i−τmi)xT(s)MTT3Mx(s)ds,−∫t−τ2it−τmi(τ2i−τmi)x˙T(s)MTU3Mx˙(s)ds,−∫t−d2it−dmi(d2i−dmi)xT(s)MTT6Mx(s)ds,−∫t−d2it−dmi(d2i−dmi)x˙T(s)MTU6Mx˙(s)ds,

we have

(44) −∫t−τ2it−τmi(τ2i−τmi)xT(s)MTT3Mx(s)ds  ≤−ξT(t)E14T3E14Tξ(t),−∫t−τ2it−τmi(τ2i−τmi)x˙T(s)MTU3Mx˙(s)ds  ≤−ξT(t)(E4−E5)U3(E4T−E5T)ξ(t),−∫t−d2it−dmi(d2i−dmi)xT(s)MTT6Mx(s)ds  ≤−ξT(t)E26T6E26Tξ(t),−∫t−d2it−dmi(d2i−dmi)x˙T(s)MTU6Mx˙(s)ds  ≤−ξT(t)(E17−E18)U6(E17T−E18T)ξ(t).

In addition, according to (8), we know that $M\dot{x}(t)=\Lambda \xi (t)$ and

(45) $$\begin{array}{c} {\left[M\dot{x}\left(t\right)\right]}^{T}J\left[M\dot{x}\left(t\right)\right]=\xi^{T}\left(t\right)\Lambda^{T}J\Lambda \xi \left(t\right), \end{array}$$

where Λ and *J* have been defined in Theorem 12.

From (3.1) and (37)–(45), we obtain

(46) ΓV(x(t),i,t)+||Mz(t)||2−δ2||ω(t)||2 ≤∑k=16ΓVk(x(t),i,t)−2ϵ1[MF1(x(t))]T[MF1(x(t))]  +2ϵ1[MF1(x(t))]TW2(1)(Mx(t))+2ϵ1(Mx(t))T  ×W1(1)T[MF1(x(t))]−2ϵ1[Mx(t)]TW1(1)TW2(1)  ×[Mx(t)]−2ϵ2[MF2(x(t−di(t)))]T  ×[MF2(x(t−di(t)))]  +2ϵ2[MF2(x(t−di(t)))]TW2(2)(Mx(t−di(t)))  +2ϵ2(Mx(t−di(t)))TW1(2)T[MF2(x(t−di(t)))]  −2ϵ2[Mx(t−di(t))]TW1(2)TW2(2)[Mx(t−di(t))]  −2ϵ3[MF3(x˙(t−τi(t)))]T[MF3(x˙(t−τi(t)))]  +2ϵ3[MF3(x˙(t−τi(t)))]TW2(3)(Mx˙(t−τi(t)))  +2ϵ3(Mx˙(t−τi(t)))TW1(3)T[MF3(x˙(t−τi(t)))]  −2ϵ3[Mx˙(t−τi(t))]TW1(3)TW2(3)[Mx˙(t−τi(t))]  +xT(t)MTLi⊗TLi⊗Mx(t)−δ2ωT(t)ω(t) ≤ξT(t)[λ1i(t)Ωi1+(1−λ1i(t))Ωi2+Θi02]ξ(t)  +ξT(t)[κ1i(t)Πi1+(1−κ1i(t))Πi2+Θi02]ξ(t).

For *τ*_*i*_(*t*)∈[*τ*_*mi*_, *τ*_2*i*_] and *d*_*i*_(*t*)∈[*d*_*mi*_, *d*_2*i*_], let

(47) λ2i(t)=τi(t)−τmiτ2i−τmi,  κ2i(t)=di(t)−dmid2i−dmi.

Then, following the above procedure, we can obtain

(48) ΓV(x(t),i,t)+||Mz(t)||2−δ2||ω(t)||2  ≤ξT(t)[λ2i(t)Ωi3+(1−λ2i(t))Ωi4+Θi02]ξ(t)   +ξT(t)[κ2i(t)Πi3+(1−κ2i(t))Πi4+Θi02]ξ(t).

For other situations, where *τ*_*i*_(*t*)∈[*τ*_*mi*_, *τ*_2*i*_], *d*_*i*_(*t*)∈[*d*_1*i*_, *d*_*mi*_], and *τ*_*i*_(*t*)∈[*τ*_1*i*_, *τ*_*mi*_], *d*_*i*_(*t*)∈[*d*_*mi*_, *d*_2*i*_], we derive (49) and (50), respectively, as

(49) ΓV(x(t),i,t)+||Mz(t)||2−δ2||ω(t)||2  ≤ξT(t)[λ2i(t)Ωi3+(1−λ2i(t))Ωi4+Θi02]ξ(t)   +ξT(t)[κ1i(t)Πi1+(1−κ1i(t))Πi2+Θi02]ξ(t),

(50) ΓV(x(t),i,t)+||Mz(t)||2−δ2||ω(t)||2  ≤ξT(t)[λ1i(t)Ωi1+(1−λ1i(t))Ωi2+Θi02]ξ(t)   +ξT(t)[κ2i(t)Πi3+(1−κ2i(t))Πi4+Θi02]ξ(t).

Therefore, with (46), (48), (49), and (50), by Lemma 11, the following inequality (51) is held for *τ*_*i*_(*t*)∈[*τ*_1*i*_, *τ*_2*i*_] and *d*_*i*_(*t*)∈[*d*_1*i*_, *d*_2*i*_] if (25) is satisfied:

(51) ΓV(x(t),i,t)+||Mz(t)||2−δ2||ω(t)||2<0.

If (26) is held, integrating the function in (51) from 0 to *∞*, then we have

(52) ∫0∞||Mz(t)||2dt<δ2∫0∞||ω(t)||2dt+V(0)≤δ2(∫0∞||ω(t)||2dt+xT(0)Yx(0)).

By Definition 8, the NCDNs (3) and (4) can reach *H*_*∞*_ cluster synchronization with a disturbance attenuation *δ*. This completes the proof.

**Remark 13**

It should be mentioned that the proposed Lyapunov functional contains some triple-integral terms. Compared with the existing ones, [39, 42] have shown that such a Lyapunov functional type is very effective in the reduction of conservatism. Besides, the information on the lower bound of the delay is sufficiently used by introducing the integral terms on [*t* − *τ*_*i*_(*t*), *t* − *τ*_1*i*_], [*t* − *τ*_1*i*_, *t*], [*t* − *τ*_2*i*_, *t* − *τ*_*mi*_], [*t* − *τ*_*mi*_, *t* − *τ*_1*i*_] and [*t* − *d*_*i*_(*t*), *t* − *d*_1*i*_], [*t* − *d*_1*i*_, *t*], [*t* − *d*_2*i*_, *t* − *d*_*mi*_], [*t* − *d*_*mi*_, *t* − *d*_1*i*_].

**Remark 14**

*H*_*∞*_ cluster synchronization of the neutral complex dynamical networks with Markovian switching is considered for the first time. The synchronization conditions are in the form of linear matrix inequalities (LMIs), which can be solved by utilizing the LMI toolbox in Matlab. The solvability of derived conditions depends not only on the attenuation level but also on the initial values of the complex networks.

In some special situations, the neutral delay may disappear and be regarded as *τ*_*i*_(*t*) ≡ 0, which can be described by the following equality and viewed as a general delayed complex dynamical network with Markovian switching:

(53) x˙(t)=Aix(t)+Bix(t−di(t))+DiF1(x(t))+EiF2(x(t−di(t)))+Hiω(t).

The following corollary is therefore given to guarantee *H*_*∞*_ cluster synchronization for this case.

**Corollary 15**

Given the transition rate matrix *Υ*, the initial positive definite matrix *Y* = *Y* ^*T*^ > 0, constant scalars *d*_1*i*_, *d*_2*i*_, and *d*_*mi*_ satisfying *d*_1*i*_ < *d*_*mi*_ < *d*_2*i*_, the NCDN systems (53) and (4) with sector-bounded condition (7) are *H*_*∞*_ cluster synchronization with a disturbance attenuation lever *δ* if there exist symmetric positive matrices *P*_*i*_ > 0, (*i* ∈ *S*), *Q*_*j*_ > 0, (*j* = 4,5, 6), *R*_*k*_ > 0, (*k* = 5,6, 7), *T*_*l*_ > 0, *U*_*m*_ > 0, and *V*_*n*_ > 0, (*l*, *m*, *n* = 4,5, 6) for any scalars *ϵ*_1_, *ϵ*_2_ > 0 such that the following linear matrix inequalities hold:

(54) $$\begin{array}{c} {\bar{\Pi }}_{i1}+\frac{1}{2}{\bar{\Theta }}_{i0}<0,\;\;{\bar{\Pi }}_{i2}+\frac{1}{2}{\bar{\Theta }}_{i0}<0,\\ {\bar{\Pi }}_{i3}+\frac{1}{2}{\bar{\Theta }}_{i0}<0,\;\;{\bar{\Pi }}_{i4}+\frac{1}{2}{\bar{\Theta }}_{i0}<0,\\ \bar{V}\left(0\right)<\delta^{2}x^{T}\left(0\right)Yx\left(0\right), \end{array}$$

where

(55) Θ¯i0=∑m=116E¯mΦ¯mE¯mT+L(✠¯)+Λ¯TJ¯Λ¯−(E¯1−E¯3)U4(E¯1T−E¯3T)−(d1iE¯1−E¯9)V4(d1iE¯1T−E¯9T)−[(dmi−d1i)E¯1−E¯12]V5[(dmi−d1i)E¯1T−E¯12T]−[(d2i−dmi)E¯1−E¯13]V6[(d2i−dmi)E¯1T−E¯13T],

where ${\bar{E}}_{i}$  {*i* = 1,2,…, 16} are block entry matrices; that is,

(15) E¯4T=[000I000000000000],J¯=R5+d1i2U4+(dmi−d1i)2U5+(d2i−dmi)2U6 +d1i44V4+(dmi2−d1i2)24V5+(d2i2−dmi2)24V6,Λ¯=(Ai⊗+Xi(1)→)E¯1T+(Bi⊗+Xi(2)→)E¯2T+Di⊗E¯14T +Ei⊗E¯15T+MHiE¯16T,✠¯=E¯1(PiDi⊗+ϵ1W1(1)T+ϵ1W2(1)T)E¯14T+E¯1PiDi⊗E¯15T +E¯1PiHiE¯16T+E¯2(ϵ2W1(2)T+ϵ2W2(2)T)E¯15T,Φ¯1=L[Pi(Ai⊗+Bi⊗+Xi(1)→+Xi(2)→)−ϵ1W1(1)TW2(1)] +∑j∈SγijPj+Q4+d1i2T4+(dmi−d1i)2T5 +(d2i−dmi)2T6+Li⊗TLi⊗,Φ¯2=−ϵ2W1(2)TW2(2),  Φ¯3=Q5−Q4,Φ¯4=Q6−Q5,  Φ¯5=−Q6,Φ¯6=R6−R5,  Φ¯7=R7−R6,  Φ¯8=−R7,Φ¯14=−2ϵ1I,  Φ¯15=−2ϵ2I,Φ¯16=−δ2I,  Φ¯m=0, (m=9,10,11,12,13),Π¯i1=−E¯10T5E¯10T−2(E¯12−E¯10)T5(E¯12T−E¯10T) −(E¯3−E¯2)U5(E¯3T−E¯2T)−2(E¯2−E¯4) ×U5(E¯2T−E¯4T)−E¯13T6E¯13T−(E¯4−E¯5) ×U6(E¯4T−E¯5T),Π¯i2=−2E¯10T5E¯10T−(E¯12−E¯10)T5(E¯12T−E¯10T) −2(E¯3−E¯2)U5(E¯3T−E¯2T)−(E¯2−E¯4) ×U5(E¯2T−E¯4T)−E¯13T6E¯13T−(E¯4−E¯5) ×U6(E¯4T−E¯5T),Π¯i3=−2E¯11T6E¯11T−(E¯13−E¯11)T6(E¯13T−E¯11T) −(E¯4−E¯2)U6(E¯4T−E¯2T)−2(E¯2−E¯5) ×U6(E¯2T−E¯5T)−E¯12T5E¯12T−(E¯3−E¯4) ×U5(E¯3T−E¯4T),Π¯i4=−E¯11T6E¯11T−2(E¯13−E¯11)T6(E¯13T−E¯11T) −2(E¯4−E¯2)U6(E¯4T−E¯2T)−(E¯2−E¯5) ×U6(E¯2T−E¯5T)−E¯12T5E¯12T−(E¯3−E¯4) ×U5(E¯3T−E¯4T).

Other notations are the same as those in Theorem 12.

**Proof**

Since *τ*_*i*_(*t*) ≡ 0, we choose the Lyapunov functional as follows:

(57) V¯(x(t),i,t)=V1(x(t),i,t)+∑k=26V¯k(x(t),i,t),

where

(58) V¯2(x(t),i,t)=∫t−d1itxT(s)MTQ4Mx(s)ds +∫t−dmit−d1ixT(s)MTQ5Mx(s)ds +∫t−d2it−dmixT(s)MTQ6Mx(s)ds,V¯3(x(t),i,t)=∫t−d1itx˙T(s)MTR5Mx˙(s)ds +∫t−dmit−d1ix˙T(s)MTR6Mx˙(s)ds +∫t−d2it−dmix˙T(s)MTR7Mx˙(s)ds,V¯4(x(t),i,t)=∫−d1i0∫t+θtd1ixT(s)MTT4Mx(s)ds dθ +∫−dmi−d1i∫t+θt(dmi−d1i)xT(s)MT       ×T5Mx(s)ds dθ +∫−d2i−dmi∫t+θt(d2i−dmi)xT(s)MT       ×T6Mx(s)ds dθ,V¯5(x(t),i,t)=∫−d1i0∫t+θtd1ix˙T(s)MTU4Mx˙(s)ds dθ +∫−dmi−d1i∫t+θt(dmi−d1i)x˙T(s)MT       ×U5Mx˙(s)ds dθ +∫−d2i−dmi∫t+θt(d2i−dmi)x˙T(s)MT       ×U6Mx˙(s)ds dθ,V¯6(x(t),i,t)=∫−d1i0∫θ0∫t+λtd1i22x˙T(s)MTV4Mx˙(s)ds dλ dθ +∫−dmi−d1i∫θ0∫t+λtdmi2−d1i22x˙T(s)MT        ×V5Mx˙(s)ds dλ dθ +∫−d2i−dmi∫θ0∫t+λtd2i2−dmi22x˙T(s)MT        ×V6Mx˙(s)ds dλ dθ.

And we define

(59) ξ¯(t)=col{Mx(t) Mx(t−di(t)) Mx(t−d1i)     Mx(t−dmi) Mx(t−d2i) Mx˙(t−d1i)     Mx˙(t−dmi) Mx˙(t−d2i) M∫t−d1itx(s)ds     M∫t−di(t)t−d1ix(s)ds M∫t−d2it−di(t)x(s)ds     M∫t−dmit−d1ix(s)ds M∫t−d2it−dmix(s)ds     MF1(x(t)) MF2(x(t−di(t))) ω(t)}.

Then we follow a similar line as in proof of Theorem 12 and obtain the result.

## 4. Numerical Examples

In this section, numerical examples are presented to demonstrate the effectiveness of the developed design on *H*_*∞*_ cluster synchronization.

**Example 1**

A four-node NCDN (3) and (4) with Markovian switching between two modes is taken into consideration; that is, *N* = 4 and *M* = 2. The parametric matrices of the NCDN are given as follows:

(60) A1=[−0.40−0.150.10−0.60],  A2=[−0.300.090.20−0.40],B1=[0.20−0.150.50−0.50],  B2=[0.310.23−0.120.17],C1=[0.280.02−0.060.11],  C2=[0.510.240.02−0.44],D1=[0.2000−0.15],  D2=[0.300−0.100.23],Ei=Fi=0, (i=1,2),G1(1)=[−0.30.10.10.10.1−0.30.10.10.10.1−0.30.10.10.10.1−0.3],G2(1)=[−0.1000.10.1−0.1000.10−0.10000.1−0.1],G1(2)=[−0.20.10.100−0.20.10.10.10−0.20.10.100.1−0.2],G2(2)=[−0.20.10.1000000.10.1−0.200.10.10−0.2],G1(3)=[00000.1−0.30.10.100.1−0.30.10.10.10.1−0.3],G2(3)=[−0.200.10.10.1−0.20.10.100000.100.1−0.2],Γ1i=Γ2i=Γ3i=[1001],  Li=[1110],            i∈S={1,2},Hk1=[01],  Hk2=[10]       (k=1,2,3,4).

The transition rate matrix is considered as follows:

(61) $$\begin{array}{c} \Upsilon =\left[\begin{array}{cc} -1 & 1\\ 2 & -2 \end{array}\right]. \end{array}$$

Furthermore, as a result of *E*_*i*_ = *F*_*i*_ = 0, only the nonlinear function *f*_1_(*x*_*k*_(*t*)) is effective and given as

(62) $$\begin{array}{c} f_{1}\left(x_{k}\left(t\right)\right)={\left[\begin{array}{cc} 0.5x_{k1}(t)-\tanh\left(0.2x_{k1}\left(t\right)\right)+0.2x_{k2}(t) & 0.95x_{k2}(t)-\tanh\left(0.75x_{k2}\left(t\right)\right) \end{array}\right]}^{T}. \end{array}$$

Then, it is easy to verify that

(63) W1(1)=[0.30.200.3],  W2(1)=[0.50.200.95].

The interval mode-dependent time-varying neutral delays and discrete delays are, respectively, assumed to be

(64) τ1(t)=0.5(1+sin3(t)),  τ2(t)=0.5(1+cos⁡3(t)),d1(t)=0.1+|sint|,  d2(t)=0.1+|cos⁡t|.

**Figure 1:**
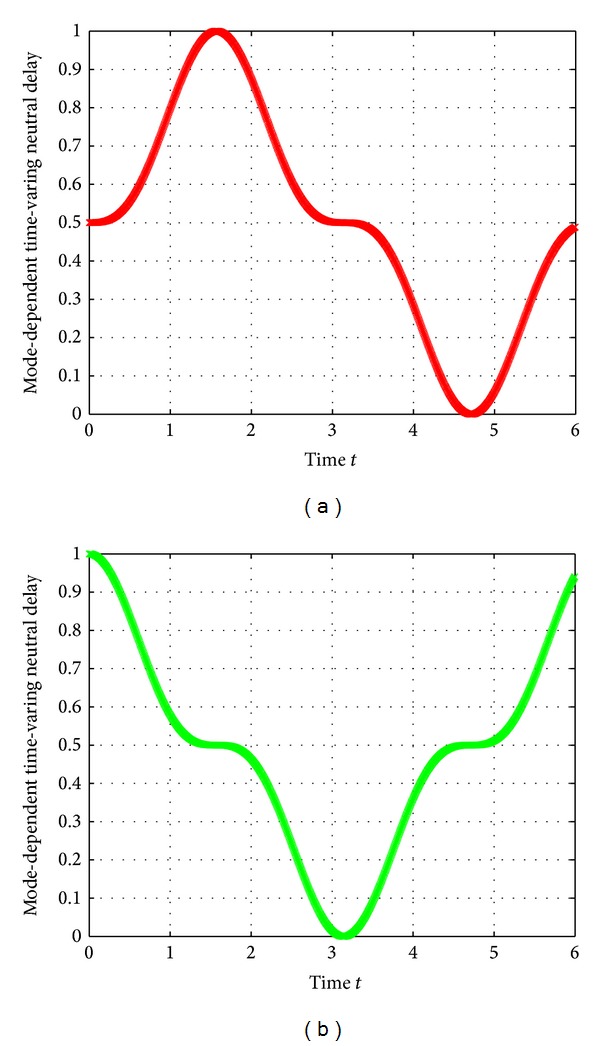
Mode-dependent time-varying neutral delays *τ*_*i*_(*t*) at mode 1 and mode 2.

**Figure 2:**
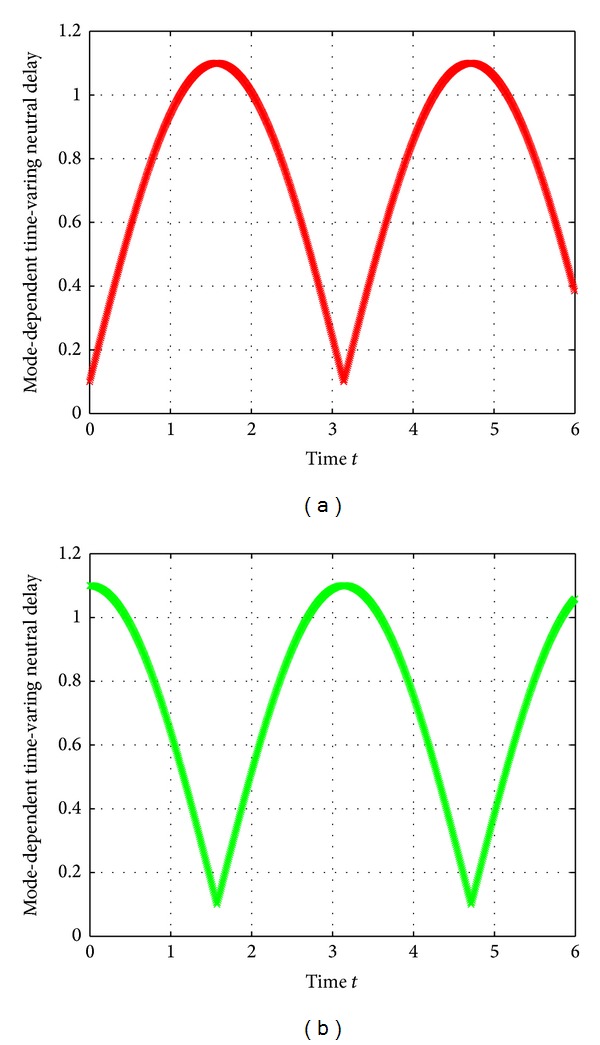
Mode-dependent time-varying retarded delays *d*_*i*_(*t*) at mode 1 and mode 2.

They are governed by the Markov process {*r*(*t*), *t* ≥ 0} and shown in Figures 1 and 2. It can be readily obtained that

(65) $$\begin{array}{c} \tau_{11}=0,\;\;\tau_{21}=1;\;\;\tau_{12}=0,\\ \tau_{22}=1;\;\;\nu_{1}=\nu_{2}=\frac{\sqrt{3}}{3},\\ d_{11}=0.1,\;\;d_{21}=1.1;\\ d_{12}=0.1,\;\;d_{22}=1.1. \end{array}$$

*H*_*∞*_ cluster synchronization of this NCDN based on the above criterion is tested. Choose *τ*_*m*1_ = 0.2, *τ*_*m*2_ = 0.3, *d*_*m*1_ = 0.4, *d*_*m*2_ = 0.5, and the initial conditions

(66) x1(s)=[0.1−0.1],  x2(s)=[0.20.1],x3(s)=[0.3−0.3],  x4(s)=[0.3−0.2],           s∈[−ς,0].

Let the disturbance attenuation level *δ* = 0.5, and let the initial positive definite matrix *Y* = 3*I*_8_. With Theorem 12, by using the Matlab LMI Toolbox, a group of matrices as a feasible solution can be obtained in the following (for simplicity, we only list the matrices for *P*_*i*_ and *Q*_*j*_, *i* ∈ *S*, *j* = 1,2,…, 6):

(67) P1=[1.78020.0659−0.00470.0028∗1.03040.0012−0.0015∗∗1.85460.0326∗∗∗1.1325],P2=[1.63720.1644−0.00420.0040∗1.45260.0033−0.0025∗∗1.37480.0727∗∗∗1.2369],Q1=[2.35890.0467−0.00190.0016∗3.13240.0016−0.0015∗∗2.10460.4326∗∗∗3.1433],Q2=[3.08110.0259−0.00340.0027∗3.32450.0029−0.0038∗∗3.64350.0037∗∗∗3.1046],Q3=[2.30420.1654−0.00030.0002∗1.63450.0018−0.0014∗∗1.86730.0756∗∗∗1.0564],Q4=[2.36320.0735−0.00110.0007∗2.04110.0134−0.0001∗∗1.77450.0542∗∗∗1.0643],Q5=[3.1822−0.0453−0.0003−0.0005∗3.331400∗∗3.2446−0.0443∗∗∗3.0418],Q6=[2.6433−0.0059−0.00500.0042∗2.30740.0014−0.0005∗∗2.04350.0926∗∗∗1.8663].

It can be concluded that this neutral complex dynamical network (NCDN) has achieved *H*_*∞*_ cluster synchronization, which illustrates the effectiveness of Theorem 12.

**Example 2**

Particularly, consider *τ*_*i*_(*t*) ≡ 0 in Example 1 and other elements are identical with Example 1. With Corollary 15, by utilizing Matlab LMI Toolbox, the LMIs (54) can be solved. Then a group of matrices as a feasible solution can be obtained as follows (for simplicity, we only list the matrices for *P*_*i*_ and *Q*_*j*_, *i* ∈ *S*, *j* = 4,5, 6):

(68) P1=[1.54330.0049−0.00060.0003∗1.02410.0018−0.0017∗∗1.03270.0034∗∗∗1.0065],P2=[1.56380.1536−0.00320.0028∗1.36740.0026−0.0011∗∗1.26550.0424∗∗∗1.0258],Q4=[2.18440.0632−0.00090.0006∗2.00870.0136−0.0001∗∗1.75490.0466∗∗∗1.0557],Q5=[3.1756−0.0346−0.0003−0.0004∗3.326700∗∗3.2338−0.0365∗∗∗3.0344],Q6=[2.6368−0.0047−0.00280.0035∗2.28660.0006−0.0003∗∗2.03370.0677∗∗∗1.8359].

It also can be proved that the complex dynamical network (CDN) has achieved *H*_*∞*_ cluster synchronization, which verifies the effectiveness of Corollary 15.

## 5. Conclusions

In this paper, *H*_*∞*_ cluster synchronization of neutral complex dynamical networks with Markovian switching is considered for the first time. By interval mode-dependent delays dividing, a new augmented Lyapunov functional containing some triple-integral terms is constructed to reduce conservativeness. Then the delay-range-dependent *H*_*∞*_ cluster synchronization criteria are obtained by the Lyapunov stability theory, integral matrix inequalities, and convex combination. Finally, numerical examples are given to illustrate the feasibility and effectiveness of the proposed result.
